# Preictal connectivity dynamics: Exploring inflow and outflow in iEEG networks

**DOI:** 10.3389/fnetp.2025.1539682

**Published:** 2025-04-28

**Authors:** Amirhossein Jahani, Camille Begin, Denahin H. Toffa, Sami Obaid, Dang K. Nguyen, Elie Bou Assi

**Affiliations:** ^1^ Centre de Recherche du Centre Hospitalier de l'Université de Montréal (CRCHUM), Montréal, QC, Canada; ^2^ Department of Neuroscience, University of Montréal, Montréal, QC, Canada; ^3^ Division of Neurosurgery, Centre hospitalier de l'Université de Montréal, Montréal, QC, Canada; ^4^ Department of Surgery, Université de Montréal, Montréal, QC, Canada

**Keywords:** intracranial EEG, inflow/outflow dynamics, seizure onset zone, preictal period, network physiology

## Abstract

**Introduction:**

Focal resective surgery can be an effective treatment option for patients with refractory epilepsy if the seizure onset zone is accurately identied through intracranial EEG recordings. The traditional concept of the epileptogenic zone has expanded to the notion of an epileptogenic network, emphasizing the role of interconnected brain regions in seizure generation. Precise delineation of this network is essential for optimizing surgical outcomes. Over the past 3 decades, several quantitative connectivity methods have been developed to study the interactions between the seizure onset zone and non-involved regions. Despite these advances, the mechanisms governing the transition from interictal to ictal periods remain poorly understood. In this study, we investigated preictal interactions between the seizure onset zone and the broader network using directed connectivity measures.

**Methods:**

We evaluated their effectiveness in identifying seizure onset zones using a multicenter intracranial EEG dataset, encompassing 243 seizures from 61 patients. Directed transfer function and partial directed coherence were used to extract connectivity matrices during 28-seconds of preictal period in patients with good surgery outcomes. Inflow and outflow metrics were computed for iEEG electrode contact annotated as seizure onset zone and the performance of each metric is assessed in disentangling these electrodes from the rest of the network.

**Results:**

We observed two distinct patterns of network connectivity preceding seizure onset. While there was an increase in inflow of information to seizure onset electrodes in one subset of patients, in the other subset, there was a prominent outflow of information from seizure onset electrodes to the rest of the network, suggesting distinct connectivity patterns associated with the seizure onset zone across patients. Further analyses showed that patients who underwent the grid/strip/depth implantation approach exhibited significantly higher area under curve (AUC) than those with electrocorticography (ECoG) or stereo-electroencephalography (sEEG) alone. Finally, examining the influence of lesional vs non-lesional neuroimaging status demonstrated that a greater proportion of high-inflow and high-outflow were lesional.

**Conclusion:**

Our findings reinforce the notion that seizure generation is driven by dynamic shifts in information flow within the brain's functional network. The preictal connectivity patterns observed --either increased inflow to the seizure onset zone or high outflow from it --underscore the network reorganization involved in epileptic transitions. These results emphasize epilepsy as a network-level phenomenon, supporting the use of network concepts for better understanding seizure dynamics and improving surgical localization strategies.

## 1 Introduction

In a subset of patients with refractory epilepsy, focal resective surgery may provide significant seizure control if the seizure onset zone (SOZ), i.e., clinically-identified electrode contacts where the first ictal changes are recorded, can be adequately identified based on intracranial EEG (iEEG) recordings in selected areas of suspected epileptogenicity (Wendling et al., 2010). In the early 2000s, the notion of the epileptogenic zone (EZ), i.e., neuroanatomical areas that are necessary and sufficient to generate epileptic seizures, as a single cortical focus fundamentally evolved to the concept of an epileptogenic network, highlighting that focal epilepsy involves a network of brain regions that interact to generate seizures (Bartolomei et al., 2017). In some cases, the epileptogenic network corresponds to a relatively restricted area of the brain, closely resembling the classical EZ concept (Rosenow and Luders, 2001). In others, the epileptogenic network could be more extensive and complex, which may explain why some seizures have discharges that rapidly or even simultaneously affect several brain regions on iEEG (Wendling et al., 2010; Bartolomei et al., 2005).

Accurately delineating the SOZ and its interactions with the rest of the network is critical for achieving improved seizure control following epilepsy surgery (Rummel et al., 2015). Over the past 3 decades, quantitative methods have been proposed to investigate these interactions (Bernabei et al., 2023). With the emergence of functional connectivity and graph-based methods, many studies explored alterations in the interaction between EZ, propagation zones and non-involved zones during interictal, preictal, ictal, and postictal periods (Li et al., 2016; Narasimhan et al., 2020; Jiang et al., 2022; Johnson et al., 2023; Paulo et al., 2022; Doss et al., 2024; Courtens et al., 2016; Van Mierlo et al., 2013; Bettus et al., 2011; Lagarde et al., 2018; Varotto et al., 2012; Krishnan et al., 2024). Notably, four main families of connectivity approaches have garnered significant attention: Granger causality (Li et al., 2016; Narasimhan et al., 2020; Jiang et al., 2022; Johnson et al., 2023; Paulo et al., 2022; Doss et al., 2024; Van Mierlo et al., 2013; Coito et al., 2019; Janca et al., 2021; Jung et al., 2011; Korzeniewska et al., 2014; Van Mierlo et al., 2011; Van Mierlo et al., 2016; Vespa et al., 2020; Wilke et al., 2010; Yang and Madsen, 2021; Bou Assi et al., 2019), information theory (Karunakaran et al., 2018; Shamas et al., 2022; Weiss et al., 2022), amplitude-based methods (Antony et al., 2013; Bernabei et al., 2022; Conrad et al., 2022; Englot et al., 2015; Fan et al., 2022; Shah et al., 2019; Tomlinson et al., 2018; Tomlinson et al., 2017; Zaveri et al., 2009), and phase-based methods (Campora et al., 2019; Fujiwara et al., 2022; Ibrahim et al., 2012; Ibrahim et al., 2013; Nissen et al., 2016; Rijal et al., 2023; Shokooh et al., 2021; Bou Assi et al., 2020). Each method offers unique insights but also presents methodological limitations. For instance, methods based on Granger causality such as the directed transfer function (DTF) and partial directed coherence (PDC) can indicate the direction of information flow between iEEG electrode contacts but are blind to non-linear interactions between or within signals. Furthermore, due to the different normalization procedures that these methods require, PDC is assumed to be more sensitive to inflow and controls for indirect connections, while DTF compromises the sensitivity to outflow and reflects both directed and non-directed connections. Although directed connectivity approaches have shown overall superior performances in disentangling SOZ contacts form the rest of the network (non-SOZ) contacts compared to non-directed approaches (Narasimhan et al., 2020; Paulo et al., 2022), variations in methodologies such as the choice of frequency band can influence the performance of these measures (Courtens et al., 2016).

The functional brain network, derived from iEEG recordings, has the potential to uncover valuable insights about underlying network’s properties and internal organization at global, local and intermediate scales (for a review please refer to (Brohl et al., 2023; Sinha et al., 2022)). Specifically, measures of integration, such as strength centrality and betweenness centrality, and metrics of segregation, such as clustering coefficient and assortativity, of the functional iEEG network have been shown to reflect alterations during preictal (Kuhnert et al., 2010; Geier et al., 2015; Rings et al., 2019a; Rings et al., 2019b) and ictal (Schindler et al., 2008; Kramer et al., 2010; Bialonski and Lehnertz, 2013) periods compared to seizure-free intervals. From the global scale network’s measures, it has been reproduced in several studies that both clustering coefficient and average shortest path length increase during seizure periods, manifesting a more segregated network.

While various approaches with differences in methodologies have been used to estimate interactions between SOZ and non-SOZ, two primary patterns consistently emerged across studies. During the ictal period, the SOZ exerts control over the network, driving it toward a hyper-synchronized state (Courtens et al., 2016; Van Mierlo et al., 2013; Lagarde et al., 2018). In contrast, during the interictal period, non-SOZ actively suppress the SOZ, providing a compelling explanation for why the epileptic brain does not seize continuously (Jiang et al., 2022; Johnson et al., 2023; Gunnarsdottir et al., 2022). This prompts important questions about the mechanisms underlying the transition from the interictal to the ictal phase, as only a few studies have explored the interaction dynamics between SOZ and non-SOZ during the preictal period (Li et al., 2016; Courtens et al., 2016)—an investigation that could offer valuable insights into the mechanisms of seizure generation. Specifically, does the non-SOZ continue to exert suppressive control (e.g., the mechanism of actively suppressing the SOZ by the rest of the network (e.g., the healthy regions) during seizure-free periods) over the SOZ up until seizure onset, or does the SOZ gradually overcome this suppression during the preictal period?

In this study, we investigated the dynamics and interactions between the SOZ and non-SOZ during the preictal period using two connectivity approaches: PDC and DTF. Furthermore, we aimed to evaluate and compare the effectiveness of these graph-based connectivity measures in distinguishing SOZ from non-SOZ contacts. The analysis was conducted on an iEEG dataset from four centers, including a total of 243 seizures from 61 patients.

## 2 Materials and methods

### 2.1 Patient selection

iEEG recordings from three datasets were used for the analysis, including one dataset from epilepsy centre of Centre Hospitalier de l'Université de Montréal (CHUM) and two publicly available datasets in standard BIDS format on OpenNeuro (Bernabei et al., 2023). The open-access datasets include ds003029, which contains iEEG recordings from National Institute of Health (NIH) and University of Maryland Medical Center (UMMC) and ds004100, which includes recordings from Johns Hopkins Hospital (JHH). All datasets include detailed clinical metadata such as implant type, surgical procedure (i.e., resection or ablation), postoperative outcome, electrode labels, and standardized coordinates. These datasets also specify which electrode contacts were targeted by surgery. Seizures onset channels were identified by board-certified epileptologists at each center independently, and the non-SOZ channels were defined as all remaining channels. In the open-access datasets (ds003029 and ds004100), SOZs were provided for each patient (though not for individual seizures), along with the channels overlapping the resection or ablation zones. For the CHUM dataset, SOZ were annotated for each seizure independently. iEEG recordings from the different centers were collected at varying sampling frequencies, ranging from 250 Hz to 2 kHz. Recordings with an original sampling rate below 500 Hz were excluded from the analysis, as this was deemed the minimum acceptable rate for reliable multivariate autoregressive modeling of iEEG segments to compute Granger causality connectivity matrices (Li et al., 2016; Van Mierlo et al., 2011; Wilke et al., 2010; Wilke et al., 2009). Additionally, iEEG recordings were excluded from the analysis if they were too short (<30 s of preictal activity), lacked information on seizure onset timing, or involved purely electrographic seizures (without clinical manifestations). All participants underwent invasive presurgical evaluations to localize SOZs and in many cases, map eloquent brain areas. Stereoelectroencephalography (SEEG), electrocortigography (ECoG) or a combination of strip, grid, and depth electrodes were placed in regions of suspected epileptogenicity. Inclusion criteria were patients with favorable surgical outcomes (Engel class I or II) and a minimum of two recorded electroclinical seizures. The results presented in this study are based on three distinct iEEG datasets (i.e., CHUM, ds003029, and ds004100) rather than being stratified by the four contributing centers.

### 2.2 Data analysis

#### 2.2.1 Preprocessing

**FIGURE 1:**
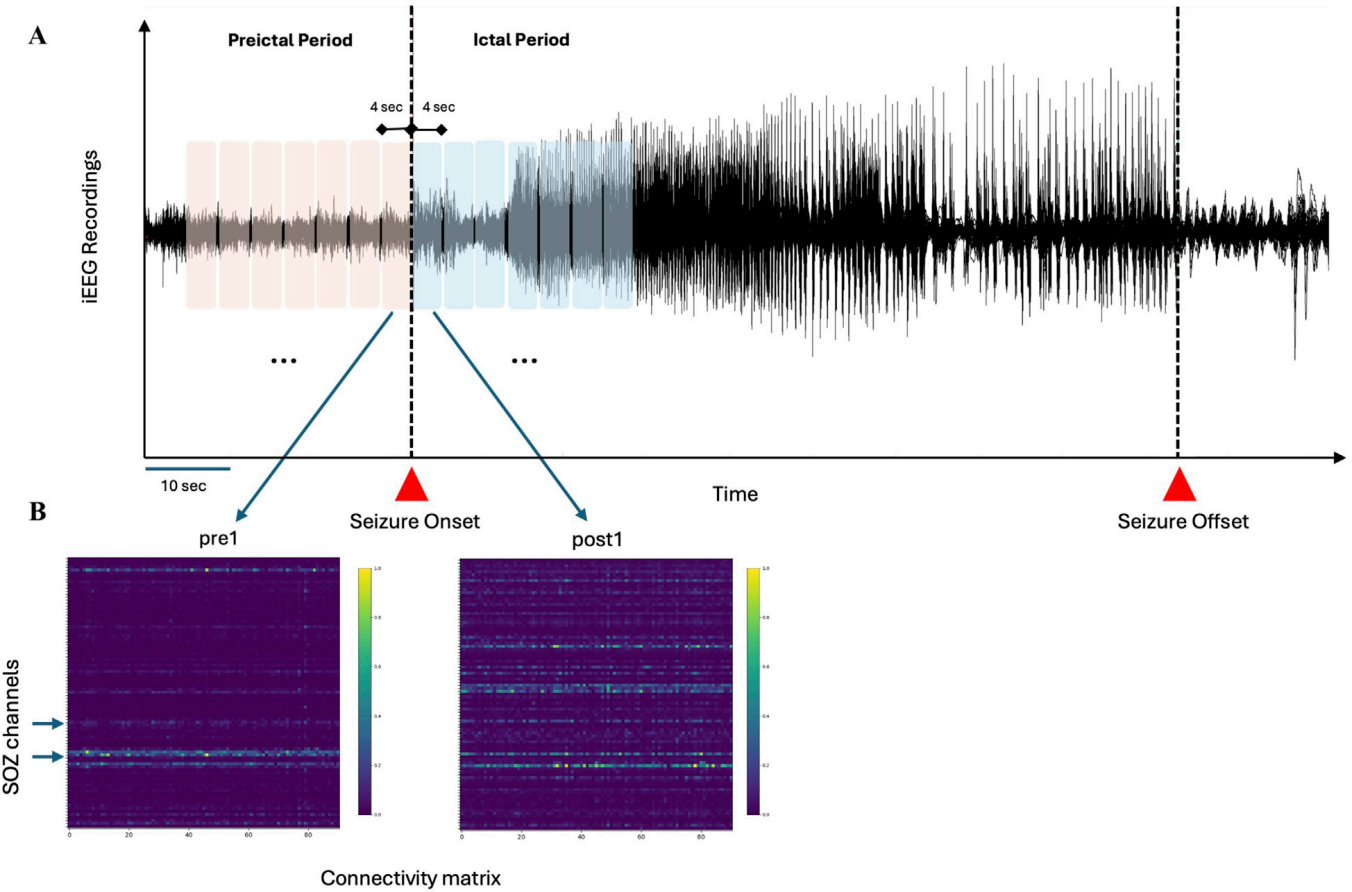
Schematic representation of the analysis pipeline **(A)** Preictal and ictal periods, totaling 28 s, are divided into seven segments of 4 s each **(B)** For each segment, connectivity matrices (PDC and DTF) are computed, with surrogate testing applied to eliminate random connections. Hypothetical SOZ channels, identified through visual inspection by epileptologists, are used to assess the performance of inflow and outflow measures in distinguishing SOZ from non-SOZ channels.

As this study does not involve neuroimaging data, a formal harmonization process was not required. However, to ensure consistency across datasets from different centres, we applied standardized preprocessing pipeline to all iEEG recordings. This included uniform filtering, artifact removal, and normalization procedures to minimize variability between datasets and maintain comparability of results. For all iEEG datasets (i.e., CHUM, ds003029, and ds004100), electrodes identified as artifactual through visual inspection by expert epileptologists were excluded from the analysis. The remaining electrodes were band-pass filtered (3–45 Hz) with a notch filter at 60 Hz and subsequently downsampled to 500 Hz in MNE-Python (Gramfort et al., 2013). Additionally, the signals were standardized using z-scoring prior to performing effective connectivity analysis (van Mierlo et al., 2018). For each seizure, we analysed 28 s of preictal iEEG recordings (before seizure onset) and 28 s of ictal iEEG recordings (following electrical seizure onset). To capture connectivity dynamics and meet quasi-stationary requirements of multivariate autoregressive models (MVAR), each 28-s period was partitioned into seven non-overlapping 4-s segments (Li et al., 2016). Schwarz’s Bayesian criterion was used to estimate model order selection during the MVAR estimation step. We observed that for each patient, the model order remained consistent across 4-s epochs within and across seizures. An overview of the data analysis pipeline is presented in Figure 1.

#### 2.2.2 Connectivity analysis

Two connectivity methods, namely, DTF and PDC, were selected to analyse preictal and ictal iEEG recordings. Connectivity analysis was performed in MATLAB using custom scripts and FieldTrip toolbox (www.fieldtriptoolbox.org) functions (Oostenveld et al., 2011).

DTF and PDC are complementary methods used to infer the direction of information flow in a network. DTF provides a global measure of how activity from one region can influence others, making it particularly relevant for understanding large-scale epileptic networks involved in seizure propagation. PDC, on the other hand, offers a more focused view, emphasizing direct, pairwise directional influences, making it well-suited for identifying the primary drivers of seizure activity within a specific brain region (Li et al., 2016; Narasimhan et al., 2020; Johnson et al., 2023; Paulo et al., 2022; Van Mierlo et al., 2013; Shokooh et al., 2021). Both DTF and PDC methods use a multivariate autoregressive modelling approach. The open-source MATLAB package ARFIT (Neumaier and Schneider, 2001; Schneider and Neumaier, 2001) was used to estimate autoregressive coefficients for each non-overlapping 4-s window. Optimal model order was defined by minimizing the Akaike information criterion (Akaike, 1974).

#### 2.2.3 Surrogate testing analysis

To assess the statistical significance of the connectivity estimates and eliminate spurious interactions between iEEG channels, a nonparametric surrogate data method was employed. In this approach, the original iEEG time series was Fourier transformed, and the phases of the Fourier coefficients were randomly shuffled to create surrogate data, disrupting temporal correlations between channels while preserving the spectral properties of the signals. The connectivity methods were then applied to the surrogate datasets, and this process was repeated 100 times to generate a distribution of connectivity values corresponding to the null hypothesis of no true interaction. Based on this distribution, a significance threshold was set at *p* = 0.05, and any connectivity links that did not exceed this threshold were considered spurious and discarded from further analysis.

#### 2.2.4 Graph analysis

Inflow and outflow were extracted from DTF and PDC connectivity matrices for each iEEG channel. These measures have demonstrated strong performance in distinguishing SOZ nodes from the non-SOZ nodes during both interictal and ictal periods (Narasimhan et al., 2020; Jiang et al., 2022; Paulo et al., 2022; Doss et al., 2024; Janca et al., 2021). We used the Brain Connectivity Toolbox (www.brain-connectivity-toolbox.net) (Rubinov and Sporns, 2010) to compute these measures.

#### 2.2.5 Performance assessment

To evaluate the performance of each graph measure (i.e., inflow and outflow), we assessed the area under the receiver operating characteristic (ROC) curve. The true positive rate was plotted against the false positive rate using the thresholded values of each normalized measure against the ground truth, defined as the seizure onset channels identified by expert epileptologists. A full threshold range from 0 to 1 was considered. The area under the ROC curve (AUC) was then computed to assess the performance of the various measures, with an AUC of 1 representing perfect classification (Burns et al., 2014).

#### 2.2.6 Statistical analysis

The normality of the inflow and outflow distributions for SOZ and non-SOZ channels was assessed using the Kolmogorov-Smirnov test. As normality was rejected, the non-parametric Wilcoxon rank-sum test was used to compare the median inflow and outflow distributions, as well as AUC values between the two patient groups. To account for multiple comparisons, Bonferroni correction was applied.

## 3 Results

### 3.1 Patient population

In total, we analyzed iEEG data from 61 patients (26 females; mean age: 34.5, range: 13–59 years) who underwent invasive monitoring for medically refractory epilepsy. The seizure duration across three datasets were high variable. Specifically, the median seizure duration in CHUM, ds004100, and ds003029 datasets are 55.80 s [IQR: 37.82–80–87], 73 s [IQR: 43–103], and 105.71 s [IQR: 86.44–158.50]. A total of 243 seizures (mean number of iEEG channels: 92.96, range: 45–176). Patient demographics are provided in Table 1.

**TABLE 1:** Presents the demographic and clinical characteristics of the 61 patients included in this study. In the CHUM iEEG recordings, seizure onset zones were annotated for each seizure. Therefore, the average number of SOZ per patient is presented in the table. Acronyms used: F (female), M (male), NIH (National Institute of Health), CHUM (the Centre hospitalier de l'Université de Montréal), JHH (Johns Hopkins Hospital), UMMC (the University of Maryland Medical Center), L (left), R (right), MTS (Mesial Temporal Sclerosis), CD (Cortical Dysplasia), MTL (Mesial Temporal Lobe), SOZ (seizure onset zone), AVE (average), MFL (Mesial Frontal Lobe).

Patients’ ID	Clinical centre	Number of seizures	Number of channels	Number of resected channels	Number of seizure onset channels	Engle score	Years of follow up	Age at surgery	Gender	Presumed epileptogenic brain region	Imaging findings	Modality
pt1	nih	4	84	n/a	10	I	3	30	F	R anterior temporal lobe	Large area of encephalomalacia in R parietal region. Smaller areas in R and L posterior temporal regions. Possible R MTS as well	ECOG
pt2	nih	3	62	n/a	8	I	3	28	F	L anterior temporal lobe	L mesial temporal sclerosis	ECOG
pt3	nih	2	97	n/a	37	I	2	45	M	R frontal lobe	normal	ECOG
pt6	nih	3	80	n/a	12	II	3	33	M	L anterior temporal lobe	nonspecific	ECOG
pt8	nih	3	59	n/a	16	I	2	25	M	R posterior temporal lobe	normal	ECOG
pt10	nih	3	55	n/a	10	II	1	44	F	L anterior temporal lobe	L MTS	ECOG
pt11	nih	4	78	n/a	24	I	1	31	M	R parietal lobe	normal	ECOG
pt13	nih	4	117	n/a	6	I	2	27	M	R parietal lobe	normal	ECOG
pt15	nih	4	71	n/a	18	I	2	59	F	L anterior temporal lobe	L MTS	ECOG
pt16	nih	3	46	n/a	6	I	2	52	F	R anterior temporal lobe, meningioma	R inferior frontal meningioma	ECOG
ummc002	ummc	3	49	n/a	10	I	4	17	M	L temporal lobe	normal, L temporal hypometabolism	ECOG
ummc005	ummc	2	48	n/a	6	I	1	47	M	R temporal lobe	Normal	ECOG
ummc008	ummc	2	50	n/a	23	I	1	49	M	R temporal lobe	R MTS, normal	ECOG
ummc009	ummc	3	45	n/a	14	I	1	36	M	R lateral temporal lobe	R posterior temporal CD	ECOG
jh05	ummc	5	85	n/a	28	I	1	n/a	n/a	R temporal lobe	n/a	ECOG
HUP065	hup	3	64	15	16	IB	n/a	36	M	temporal	lesional	ECOG
HUP070	hup	5	63	23	10	IB	n/a	33	M	frontal pole	non-lesional	ECOG
HUP074	hup	5	114	59	6	IC	n/a	25	F	temporal	lesional	ECOG
HUP082	hup	5	86	43	13	IA	n/a	56	F	temporal	lesional	ECOG
HUP087	hup	2	84	5	12	ID	n/a	24	M	frontal	lesional	ECOG
HUP088	hup	3	54	7	8	ID	n/a	35	F	temporal	lesional	ECOG
HUP089	hup	4	94	10	3	IB	n/a	29	M	temporal	lesional	ECOG
HUP094	hup	3	83	3	3	IB	n/a	48	F	temporal	non-lesional	ECOG
HUP097	hup	5	92	16	7	ID	n/a	39	F	temporal	non-lesional	ECOG
HUP105	hup	2	55	4	9	IA	n/a	39	M	temporal	lesional	ECOG
HUP106	hup	3	115	10	5	IB	n/a	45	F	temporal	non-lesional	ECOG
HUP107	hup	5	117	23	22	IA	n/a	36	M	temporal	non-lesional	ECOG
HUP111	hup	5	101	7	13	IB	n/a	40	F	temporal	non-lesional	ECOG
HUP116	hup	3	50	5	8	IA	n/a	59	F	MTL	lesional	SEEG
HUP117	hup	3	49	3	7	IA	n/a	39	M	temporal	lesional	SEEG
HUP123	hup	4	117	24	7	IA	n/a	36	M	temporal	lesional	ECOG
HUP126	hup	4	125	9	2	IA	n/a	26	F	MTL	non-lesional	ECOG
HUP130	hup	5	120	3	2	IB	n/a	46	F	MFL	non-lesional	SEEG
HUP139	hup	3	73	11	8	IA	n/a	20	M	parietal	lesional	SEEG
HUP140	hup	3	86	6	4	IB	n/a	47	F	MTL	non-lesional	SEEG
HUP141	hup	5	113	13	13	IC	n/a	30	M	MTL	non-lesional	SEEG
HUP142	hup	3	108	10	15	ID	n/a	30	M	MTL	lesional	SEEG
HUP144	hup	5	111	15	6	ID	n/a	31	M	temporal	lesional	SEEG
HUP146	hup	3	122	7	11	IA	n/a	16	M	temporal	non-lesional	SEEG
HUP148	hup	5	101	11	25	IA	n/a	23	M	temporal	lesional	SEEG
HUP150	hup	5	89	1	8	IB	n/a	17	M	insular	lesional	SEEG
HUP157	hup	5	164	6	13	IB	n/a	25	M	MTL	non-lesional	SEEG
HUP160	hup	3	102	13	21	IA	n/a	45	F	temporal	non-lesional	SEEG
HUP163	hup	3	156	8	6	ID	n/a	42	F	MTL	non-lesional	SEEG
HUP164	hup	3	176	3	9	ID	n/a	34	F	MTL	lesional	SEEG
HUP177	hup	3	172	18	13	IA	n/a	42	F	temporal	non-lesional	SEEG
HUP180	hup	5	111	5	8	IA	n/a	28	F	frontal	lesional	SEEG
HUP185	hup	5	113	9	6	IA	n/a	38	M	MTL	lesional	SEEG
pt1	chum	11	97	6	AVE = 6	I	n/a	49	M	R parietal lobe	non-lesional	Grid/strips and depth
pt2	chum	5	104	12	AVE = 8.6	I	n/a	32	F	R fronto-parietal	non-lesional	Grid/strips and depth
pt7	chum	9	61	20	AVE = 7.1	I	n/a	29	F	L frontal plus (=fronto-temporo-insular)	non-lesional	Grid/strips and depth
pt9	chum	4	105	6	AVE = 8.5	I/II	n/a	18	F	L insular-opercular	non-lesional	Grid/strips and depth
pt11	chum	7	91	7	AVE = 6.4	I	n/a	35	M	R frontal	cortical atrophy	Grid/strips and depth
pt12	chum	3	88	12	AVE = 4.6	I	n/a	46	M	L insular plus (insulo-opercular)	non-lesional	Grid/strips and depth
pt14	chum	4	73	6	AVE = 14.5	I	n/a	23	M	L fronto-parietal	non-lesional	Grid/strips and depth
pt16	chum	5	101	21	AVE = 3.8	II	n/a	29	M	R-combined temporal plus and generalized epilepsy (combined temporo-insular and generalized epilepsy)	hippocampal sclerosis	Grid/strips and depth
pt17	chum	4	114	25	AVE = 1	II	n/a	22	M	R frontal	non-lesional	Grid/strips and depth
pt21	chum	5	111	2	AVE = 6.8	II	n/a	36	M	L frontal plus (=fronto-insular)	no clearly reliable lesion	Grid/strips and depth
pt22	chum	5	108	25	AVE = 4.2	I	n/a	34	M	R frontal	non-lesional	Grid/strips and depth
pt23	chum	2	99	7	AVE = 8.5	II	n/a	40	F	temporal	periventricular nodular heterotopia	Grid/strips and depth
pt25	chum	3	113	7	AVE = 1.3	I	n/a	19	F	L parieto-occipital	focal CD	Grid/strips and depth

### 3.2 Per-patient analysis

We found that patients in each dataset could be categorized into two groups during the preictal period: those with high inflow to SOZ channels and those with high outflow from SOZ channels. We observed a substantial difference in the distribution of classified patients across these datasets. Notably, 77% of patients in the CHUM dataset, 30% in the ds004100 dataset, and 20% in the ds003029 dataset could be classified into one of these two groups. The summary of results is presented in Table 2.

**TABLE 2:** This table summarizes the key findings of this study. Median values of AUC and the interquartile range (IQR) are presented for each group. The following symbols are used in the table: ↑≡ Higher values in SOZ compared to non-SOZ. ≈≡ No significant different was observed.

Patient Group	Inflow (SOZ vs non-SOZ)	Outflow (SOZ vs non-SOZ)	AUC (PDC-inflow)	AUC (PDC-outflow)	Key Observation
Inflow-Dominant	↑ SOZ (higher inflow)	≈ (no significant difference)	0.57 [IQR: 0.51–0.64]	0.46 [IQR: 0.40–0.51]	SOZ receive more information
Outflow-Dominant	≈ (no significant difference)	↑ SOZ (higher outflow)	0.48 [IQR: 0.42–0.55]	0.58 [IQR: 0.52–0.65]	SOZ distribute more information
Pooled Dataset	Mixed trend	Mixed trend	0.49 [IQR: 0.44–0.56]	0.51 [IQR: 0.45–0.57]	No dominant pattern when pooling all datasets

#### 3.2.1 High inflow to SOZ channels during the preictal period

**FIGURE 2:**
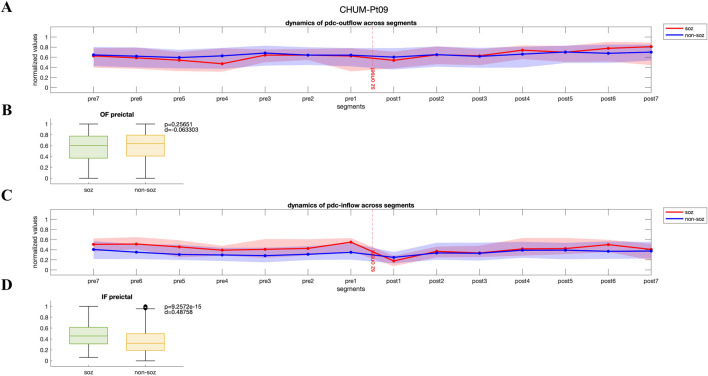
**(A, C)** Dynamics of PDC-based inflow and outflow for SOZ and non-SOZ channels over 28 s during the preictal and ictal periods is presented for pt09 from CHUM dataset **(B, D)** Although no significant difference is observed in the median outflow between SOZ and non-SOZ channels (p = 0.26, Wilcoxon rank-sum test; Cohen’s d effect size = 0.063), the median inflow to SOZ channels is significantly higher than that of non-SOZ channels (p = 9.2572e-15, Wilcoxon rank-sum test; Cohen’s d effect size = 0.49). IF: inflow; OF: outflow.

For a subset of patients in each dataset, we observed a distinct pattern where both PDC-based and DTF-based inflow of information to the SOZ channels was consistently higher than that to the non-SOZ channels in preictal period (p < 0.05, Wilcoxon rank-sum test). In Figures 2A, C, dynamics of PDC-derived normalized outflow and inflow of SOZ and non-SOZ channels during 28 s of preictal and ictal periods are plotted from Patient Pt09 (CHUM dataset), respectively. Although no significant difference is observed between the median outflow of SOZ and non-SOZ channels (p = 0.26, Wilcoxon rank-sum test; Figure 2B), the normalized inflow values for the SOZ channels rise significantly as the seizure approaches (p = 9.2572e-15, Wilcoxon rank-sum test; Figure 2D; DTF-based outflow dynamics for the same patient is shown in Supplementary Figure S1). Across the group of patients showing this pattern, the normalized median PDC-based and DTF-based inflow to SOZ channels during the seven segments of the preictal period was 0.44 [IQR: 0.26–0.61] and 0.61 [IQR: 0.44–0.76], compared to 0.32 [IQR: 0.16–0.50] and 0.56 [IQR: 0.41–0.71] for non-SOZ channels, respectively. This trend suggests that in these patients, the SOZ channels are highly influenced by the surrounding network leading up to seizure onset.

#### 3.2.2 High outflow from SOZ channels during the preictal period

**FIGURE 3:**
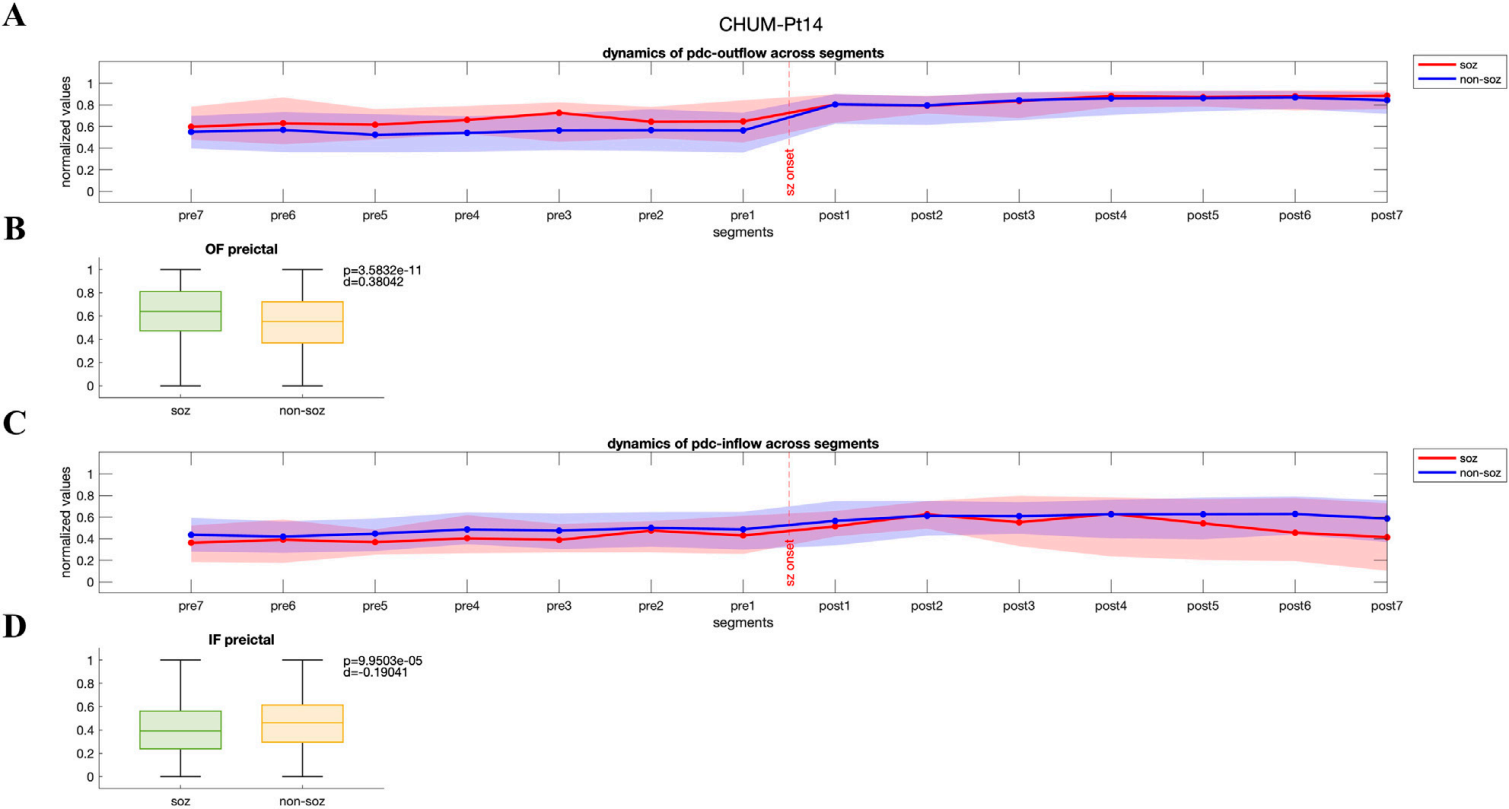
**(A, C)** Dynamics of PDC-based inflow and outflow for SOZ and non-SOZ channels over 28 s during the preictal and ictal periods is presented for pt14 from CHUM dataset **(B, D)** Statistical analysis reveals that the median outflow from SOZ channels is significantly higher than from non-SOZ channels (p = 3.58e-11, Wilcoxon rank-sum test; Cohen’s d effect size = 0.38), while the median inflow to non-SOZ channels is significantly higher than to SOZ channels (p = 9.95e-5, Wilcoxon rank-sum test, Cohen’s d effect size = 0.19). IF: inflow; OF: outflow.

In contrast, another group of patients displayed a pattern where both PDC-based and DTF-based outflow from the SOZ channels to the rest of the network was significantly higher during preictal period (p < 0.05, Wilcoxon rank-sum test), potentially contributing to seizure generation and propagation. In Figures 3A, C, PDC-derived normalized values of outflow and inflow of SOZ and non-SOZ channels during 28 s of preictal and ictal periods are illustrated from Patient Pt14 (CHUM dataset), respectively. While outflow from the SOZ channels significantly increases during preictal segments and remains elevated up to the seizure onset (p = 3.58e-11, Wilcoxon rank-sum test; Figure 3B), the median inflow to non-SOZ channels is significantly higher than to SOZ channels (p = 9.95e-5, Wilcoxon rank-sum test; DTF-based outflow dynamics for the same patient are shown in Supplementary Figure S2). For these patients, the median PDC-based and DTF-based outflow from SOZ channels during the preictal period was 0.67 [IQR: 0.52–0.80] and 0.28 [IQR: 0.10–0.47], while the non-SOZ channels showed an outflow of 0.61 [IQR: 0.44–0.76] and 0.18 [IQR:0.02–0.40], respectively.

### 3.3 Grouping of patients based on observed patterns

#### 3.3.1 Overall AUC performances for inflow and outflow measures

**FIGURE 4:**
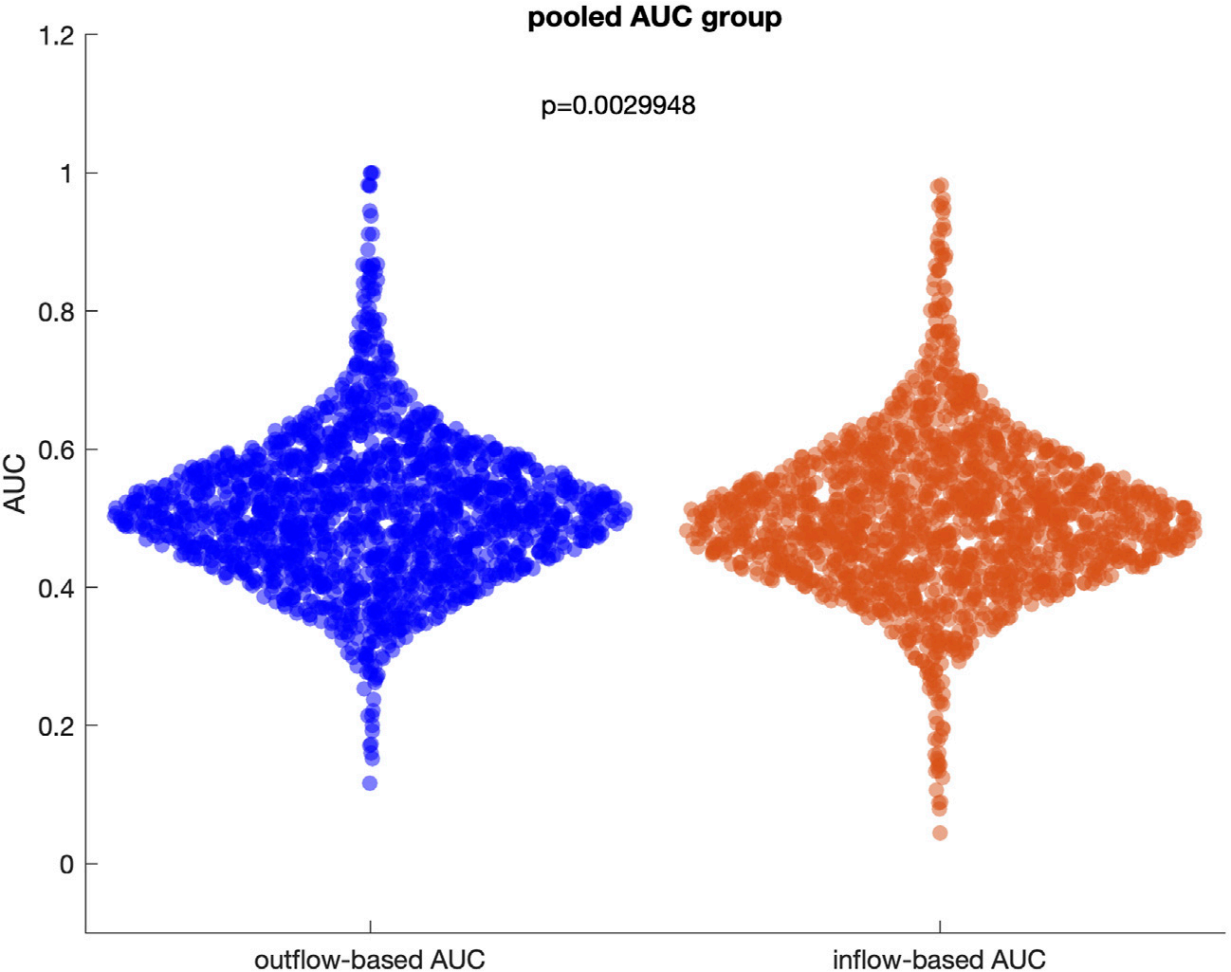
Distribution of outflow-based and inflow-based AUC values when all patients and seizures are pooled. The median of two distributions is close to 0.5, representing a random guess.

The initial analysis focused on evaluating the overall performance of inflow and outflow measures in disentangling SOZ channels from non-SOZ channels across all patients. When pooling data from the entire datasets (61 patients with 243 seizures), the ability of these measures to dissociate SOZ from non-SOZ channels was limited. For inflow measure, the median pooled AUC for separating SOZ and non-SOZ channels was 0.49 [IQR: 0.44–0.56], indicating suboptimal performance. Similarly, the median pooled AUC for outflow measures was 0.51 [IQR: 0.45–0.57], further highlighting the challenge in identifying a robust and consistent pattern across diverse patient population (Figure 4). These results suggest that, when combining all patients without accounting for individual connectivity patterns, inflow and outflow measures alone might not be sufficient to reliably disentangle SOZ from non-SOZ channels during the preictal period.

#### 3.3.2 AUC performances after grouping patients by connectivity patterns

To further explore the potential of inflow and outflow measures for the identification of SOZ channels, we regrouped patients based on the two dominant patterns observed in the network dynamics analysis: high inflow to SOZ channels and high outflow from SOZ channels.

##### 3.3.2.1 Inflow-dominant group

In patients showing high inflow to SOZ channels during the preictal period (16 patients with 66 seizures), the AUC for inflow measures slightly improved. The median PDC and DTF inflow-derived AUC for this subgroup were 0.57 [IQR: 0.51–0.64] and 0.54 [IQR: 0.48–0.61], respectively. Moreover, we observed that the median PDC and DTF outflow-derived AUC for this subgroup were 0.48 [IQR: 0.42–0.55] and 0.48 [IQR: 0.43–0.53], respectively. This suggests that inflow-based metrics could be more effective at dissociating SOZ from non-SOZ channels when this pattern is present, regardless of the connectivity method used.

##### 3.3.2.2 Outflow-dominant group

In patients where high outflow from SOZ channels to the rest of the network was observed during the preictal period (7 patients with 31 seizures), the outflow measures also showed improved performance. The median PDC and DTF outflow-derived AUC in this group increased to 0.58 [IQR: 0.52–0.65] and 0.59 [IQR: 0.54–0.70], respectively. Furthermore, the median PDC and DTF inflow-derived AUC for this subgroup were 0.46 [IQR: 0.40–0.51] and 0.46 [IQR: 0.40–0.53], respectively. This indicates that outflow dynamics could more reliably separate SOZ from non-SOZ channels in these patients.

#### 3.3.3 Comparative AUC analysis

**FIGURE 5:**
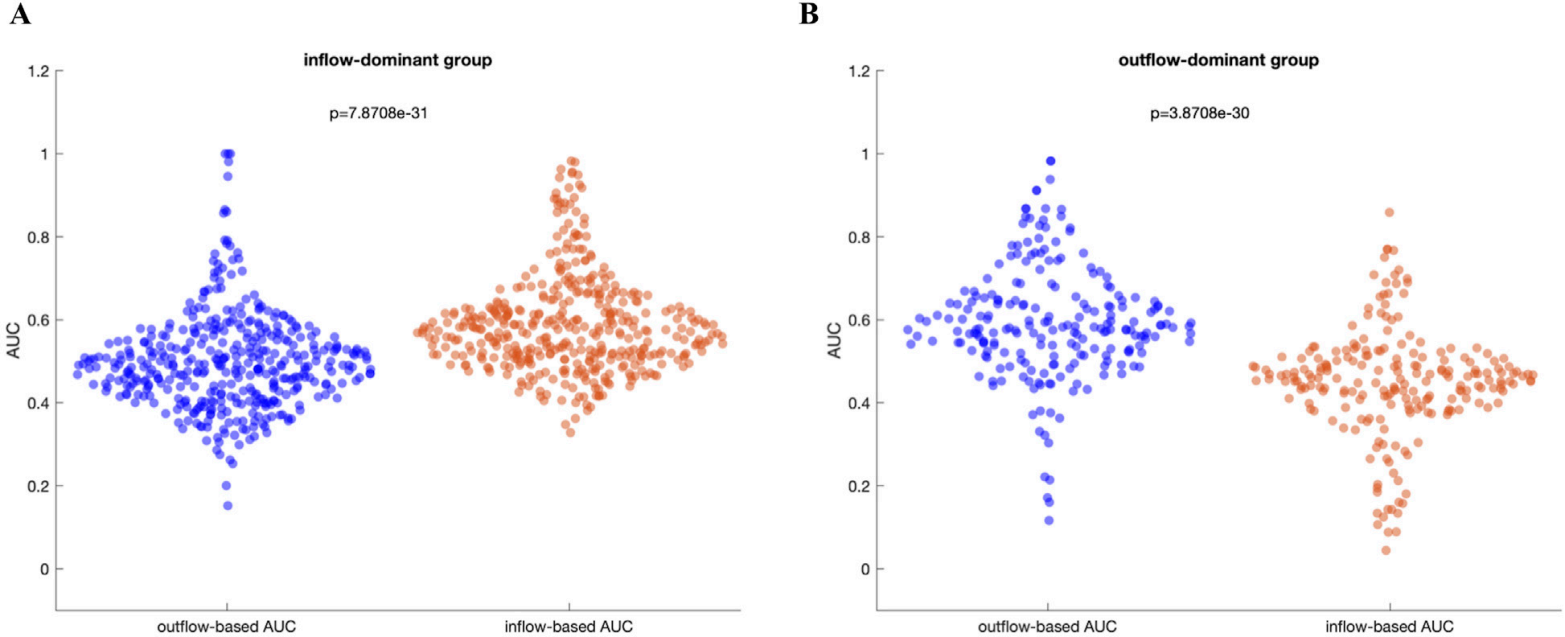
**(A)** Inflow-dominant group: In this group, the median AUC values based on inflow are significantly higher than those based on outflow (p = 7.87e-31), Wilcoxon rank-sum test **(B)** Outflow-dominant group: In this group, the median AUC values based on outflow are significantly higher than those based on inflow (p = 3.87e-30, Wilcoxon rank-sum test).

**FIGURE 6:**
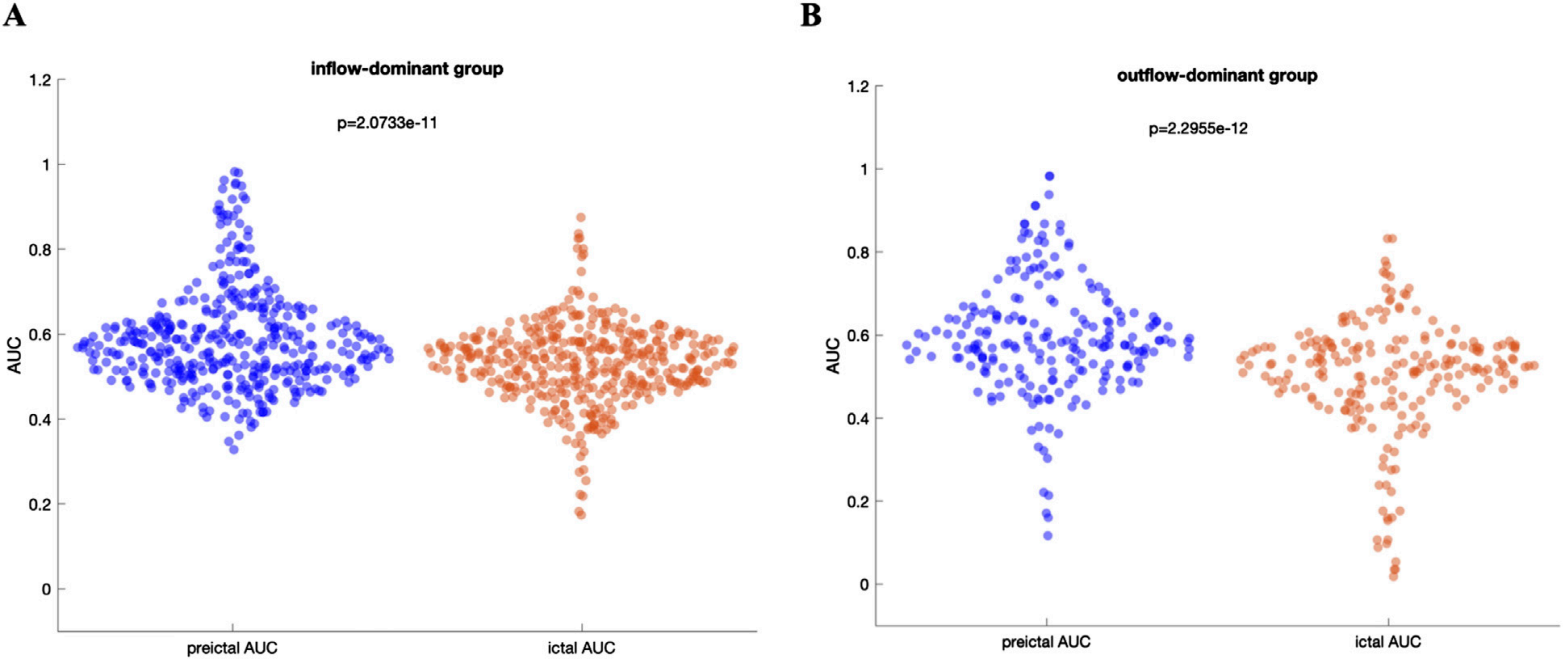
**(A)** Distribution of AUC values for the inflow-dominant group, comparing preictal and ictal periods. The preictal period shows a higher median AUC, highlighting an enhanced ability to identify SOZ channels compared to the ictal period **(B)** Distribution of AUC values for the outflow-dominant group, similarly, showing elevated median AUC values in the preictal period. Both panels underscore the improved performance in SOZ identification during the preictal phase across distinct network flow patterns, with statistically significant differences evident between preictal and ictal phases for each group.

Figure 5 presents a comparative analysis between the inflow-based AUC and outflow-based AUC values in both groups. The inflow-dominant patients consistently showed higher AUC values for inflow measures during the preictal period (p < 0.05, Wilcoxon rank-sum test), while the outflow-dominant patients exhibited improved AUC for outflow measures (p < 0.05, Wilcoxon rank-sum test). This suggests that grouping patients based on observed connectivity patterns can enhance the distinguishing power of these network measures. Interestingly, the observed improvement was preserved when DTF was used to extract inflow-based and outflow-based AUC values in both groups (Supplementary Figure S3). Comparative analysis of AUC values between the preictal and ictal periods for both inflow-dominant and outflow-dominant groups reveals that the preictal period consistently yields significantly higher median AUC values than the ictal period. This finding suggests improved performance in identifying SOZ channels when focusing on the preictal phase, with statistical significance demonstrated for both groups (p = 2.07e-11, Wilcoxon rank-sum test, for the inflow-dominant group and p = 2.29e-12, Wilcoxon rank-sum test, for the outflow-dominant group). Figures 6A, B illustrate the distribution of AUC values within each group, showcasing the marked difference in SOZ identification potential between these phases.

#### 3.3.4 The effect of number of implanted electrodes on connectivity classification categories

We conducted a correlation analysis to assess the potential influence of the distance between electrodes and number of implanted electrodes on our findings. Specifically, we observed no significant difference in the number of implanted electrodes between high-inflow and high-outflow groups (Supplementary Figure S6; p = 0.80, Wilcoxon rank-sum test; Cohen d effect size = 0.055).

#### 3.3.5 The effect of implantation approaches on connectivity classification categories

We analyzed the distribution of implantation approaches among patients and their association with connectivity classification. We observed that larger number of patients who underwent a combined implantation approach (grid/strip/depths) were classified in either the high inflow or high outflow groups (Table 3; Supplementary Figure S1). Additionally, we compared AUC values across the three implantation approaches (ECoG, sEEG, and combination of grid/strip/depth electrodes) and found that patients with a combined grid/strip/depth implantation exhibited significantly higher AUC values than those with either ECoG or sEEG alone. This pattern was consistently observed in both high inflow and high outflow groups (Supplementary Figure S8).

**TABLE 3:** This table represents the percentage of classified patients in each group (i.e., high inflow and high outflow) with respect to the implantation approach. Three approaches are identified to be used across three datasets, including ECoG, SEEG, and combination of grid/strip/depth electrodes.

Type of iEEG implantation approach	High inflow	High outflow
ECoG	20%	4%
SEEG	25%	8%
A combination of grid/strip/depth electrodes	25%	16%

These findings highlight the importance of considering implantation methodology when interpreting connectivity analyses and reinforce the notion that a hybrid implantation strategy may provide a more comprehensive assessment of epileptogenic networks.

#### 3.3.6 The effect of neuroimaging findings on connectivity classification categories

We examined whether lesional vs non-lesional status influenced our results. Among patients classified as high inflow or high outflow, we observed that a higher number of patients were lesional, as indicated by imaging findings (Supplementary Figure S9). Additionally, statistical comparisons between lesional and non-lesional groups revealed that within the high inflow group, lesional patients exhibited significantly higher AUC values, suggesting stronger discriminability in this subgroup (Supplementary Figure S10A). However, in the high outflow group, no significant difference was observed between lesional and non-lesional patients (Supplementary Figure S10B).

## 4 Discussion

### 4.1 Summary of key findings

The analysis of iEEG recordings across three datasets from four different institutions highlighted two distinct patterns of network connectivity preceding seizure onset, with implications for understanding seizure dynamics. In one subset of patients, there was an increase in inflow of information to SOZ channels during the preictal period, suggesting that these channels are influenced by surrounding areas before seizures. Our analysis revealed no significant difference in the number of implanted electrodes between patients classified into high-inflow and high-outflow groups. We found that patients who underwent a combined implantation approach (grid/strip/depths) were more frequently classified in either the high inflow or high outflow groups. Notably, these patients exhibited significantly higher AUC values compared to those with ECoG or sEEG alone, a pattern consistently observed across both connectivity groups. Additionally, we examined the influence of lesional vs non-lesional status and found that a greater proportion of high-inflow and high-outflow patients were lesional. Within the high inflow group, lesional patients demonstrated significantly higher AUC values, indicating stronger discriminability in this subgroup. However, in the high outflow group, AUC values did not significantly differ between lesional and non-lesional patients. While these findings suggest distinct preictal network dynamics, we do not claim that they directly explain seizure initiation mechanisms. Instead, they highlight variability in how SOZ and non-SOZ regions interact before seizure onset, which may reflect different underlying susceptibilities to ictogenesis. By refining our presentation, we aim to provide a clearer link between these network interactions and seizure localization without overinterpreting their mechanistic implications.

We observed that 77% of patients in the CHUM dataset, 30% in the ds004100 dataset, and 20% in the ds003029 dataset could be classified into one of these two groups. However, the overall performance of inflow and outflow measures in distinguishing SOZ from non-SOZ channels was limited when all patients were pooled together, as demonstrated by the low median AUC values. By regrouping patients based on their dominant connectivity patterns (either high inflow or high outflow), the performance of these measures improved, with both inflow and outflow metrics showing higher, although still modest, AUC values in their respective patient subgroups. This suggests that personalized connectivity patterns may enhance the ability to identify SOZ channels and underscores the need for patient-specific analyses when using network measures to analyze seizure onset and propagation.

In this study, we evaluated connectivity measures on a comprehensive dataset comprising three different sources, including one local CHUM dataset and two open-access datasets and one local CHUM dataset. This approach allows for a realistic estimation of the performance of connectivity measures in identifying the SOZ. By including multiple datasets with varying structures—such as patient-level versus seizure-level SOZ annotations—we aimed to capture a wide array of seizure dynamics and preictal patterns. This diversity is critical for evaluating the robustness of connectivity-based methodologies, as it mitigates potential biases introduced by dataset-specific patterns or patient selection criteria.

Our results demonstrate that both PDC-based and DTF-based approaches consistently revealed significant differences between SOZ and non-SOZ regions in the preictal period, regardless of whether the patients exhibited inflow-dominant or outflow-dominant connectivity patterns. Despite the conceptual and mathematical distinctions between these two methods—PDC focusing on the direct influence of regions and being more sensitive to inflow dynamics, while DTF accounting for both direct and indirect connections and having the tendency to capture subtleties in outflow—our findings suggest that these approaches can converge in identifying key network dynamics. This convergence underscores the robustness of both methods in capturing meaningful connectivity patterns during the preictal period.

### 4.2 Comparison to existing literature

A collective body of work highlights a growing consensus that the analysis of changes in network connectivity during interictal, preictal, and ictal periods, both in human patients and animal models, can contribute to improve our understanding of the mechanisms of seizure initiation (Li et al., 2016; Johnson et al., 2023; Doss et al., 2024; Van Mierlo et al., 2013).

Despite some contradictory findings in the literature (Lagarde et al., 2022), most recent studies using functional directed connectivity and other advanced approaches support two main hypotheses regarding the interaction between SOZ and non-SOZ regions. First, during the interictal period, SOZ regions are suppressed by non-SOZ regions, indicated by high inflow to SOZ regions (Johnson et al., 2023; Doss et al., 2024). Second, during the ictal period, SOZ regions act as drivers, exhibiting high outflow toward non-SOZ regions (Van Mierlo et al., 2013; Van Mierlo et al., 2016). Interestingly, although the preictal period likely holds critical insights into the mechanisms of seizure generation, only a limited number of studies have explored the dynamics of information flow between SOZ and non-SOZ regions during this crucial time frame. This gap in the literature underscores the need for further investigation into preictal connectivity patterns to better understand seizure initiation.

Li et al. (2016) explored the effectiveness of PDC-based graph measures, including indegree, outdegree, and betweenness centrality, in localizing the epileptogenic zone during the preictal period in seven patients with 21 seizures. They observed that highly connected nodes appear immediately before seizure onset, with information flowing into channels identified by epileptologists. Indegree proved highly effective across all seven patients, with a median AUC of 0.93 [IQR: 0.89–0.97], while outdegree and betweenness centrality showed only partial effectiveness, demonstrating a broader range of AUC values and considerable individual variation.

Building upon these findings, Sumsky and Greenfield (2022)further emphasized the significance of the preictal period in revealing the underlying mechanisms of seizure generation. Using iEEG data from 20 patients, the authors applied a novel multiple input, single output state-space model to create directed network graphs. Their analysis showed that highly connected nodes in the SOZ displayed increased connectivity about 37 s before seizure onset, and these changes propagated to non-SOZ regions roughly 8 s before the seizure began, pointing to distinct pre-seizure network dynamics.

In another related study, Krishnan et al. (2024) examined the spatiotemporal dynamics of seizure propagation in 10 patients with drug-resistant focal epilepsy using stereoelectroencephalography and ictal single-photon emission computed tomography. Their work revealed that the EZ exhibits significant outflow to other brain regions during both preictal and ictal phases. Notably, hypoperfused regions demonstrated enhanced connectivity with the EZ during seizure evolution, suggesting that these regions may play a critical role in modulating seizure activity and further illustrating the complexity of seizure propagation.

Complementing these human studies, Broggini et al. (2016) investigated preictal synchrony between the hippocampus and medial prefrontal cortex in a rat model of temporal lobe epilepsy induced by perforant path stimulation (Broggini et al., 2016). The authors hypothesized that alterations in theta-band oscillatory activity between these regions might precede seizures and contribute to our understanding of epileptogenesis. Their results revealed a significant increase in theta coherence between the hippocampus and medial prefrontal cortex before seizure onset, indicating heightened synchrony during this period. Furthermore, the coupling between hippocampal theta and medial prefrontal cortex gamma oscillations increased prior to seizures, with Granger causality analysis suggesting that hippocampal networks drive this synchrony. These findings underscore the potential of preictal theta coherence as a biomarker for predicting seizures and provide insights into the network dynamics of temporal lobe epilepsy.

While current study is used metrics of network theory to study dynamics of epileptic brain during preictal period, recent evidence shed light on the applicability of other innovative approaches to study alteration of SOZ and EZ (Bernabei et al., 2023; Bernabei et al., 2022). Specifically, Bernabei et al. (2022) constructed a normative iEEG atlas by retrospectively aggregating more than 5,000 iEEG channels from 166 subjects with mesial temporal lobe. They showed that normalized spectral power and coherence across frequency band could serve as biomarkers in distinguishing SOZ from non-SOZ, with connectivity abnormalities being more effective than univariate spectral power metrics (Bernabei et al., 2022).

The proportion of patients with each implantation type differs across datasets, which may explain the observed variations in classification distribution. Specifically, 77% of patients in the CHUM dataset, 30% in the ds004100 dataset, and 20% in the ds003029 dataset were classified into one of these two groups. As shown in Table 1, all patients in the CHUM dataset were implanted with a combined grid/strip/depth implantation approach, which aligns with the observed superiority of this implantation method in distinguishing SOZ from non-SOZ. Furthermore, Bernabei et al. (2021) showed that the implantation approach may affect the distinguishability of strength connectivity metrics in classifying nodes (iEEG electrode contacts) as resected or non-resected (Bernabei et al., 2021). Our analysis is consistent with this hypothesis and suggest that a combined grid/strip/depth implantation may provide a higher distinguishability of strength metrics, as demonstrated by the significant difference between AUC values of three different implantation approaches.

### 4.3 Neural correlates of sink and source hypotheses

Seizures have traditionally been linked to an imbalance between excitatory and inhibitory mechanisms, where glutamatergic neuronal populations are primarily involved in initiating focal seizures and driving the network towards hyper-synchronization. This hypothesis is supported by observations that interictal spikes are characterized by increased neuronal firing followed by reduced excitability. Directed connectivity analysis, such as PDC, in patients with drug-resistant epilepsy has shown that during resting state, connectivity towards SOZ increases (i.e., high inflow) while connectivity outward from SOZ decreases (i.e., low outflow) (Johnson et al., 2023; Doss et al., 2024). Building on these findings, it has been proposed that the transition from interictal activity to seizures is driven by a reduction in inhibition, favoring enhanced excitation (de Curtis and Avanzini, 2001). Aligned with this hypothesis, our observations suggest that as the seizure onset approaches, non-SOZ may lose their inhibitory influence over SOZ, potentially manifesting as increased outflow from SOZ in the outflow-dominant group.

Emerging evidence has further enriched this perspective by highlighting the key role for GABAergic interneurons in seizure generation. Under certain pathological conditions, such as focal seizures, GABAergic signaling-traditionally considered inhibitory-may instead exert a depolarizing effect (Ziburkus et al., 2006). Studies have demonstrated that optogenetic activation of GABAergic interneurons can induce seizure-like events, while blocking GABAergic signaling often prevents seizure initiation (Chang et al., 2018). In light of these findings, our results in the inflow-dominant group revealed that SOZ exhibited increased inward connectivity (i.e., high inflow) during the 28 s preceding seizure onset. This further supports the idea that both excitatory and inhibitory networks, including GABAergic signaling, play a complex role in driving the pre-seizure dynamics. Although excitatory/inhibitory mechanisms could not be directly correlated with outflow/inflow of information measured by Granger causality methods, these lines of evidence suggest that the high inflow to SOZ channels during the preictal period, observed in the current study and some previous studies (Li et al., 2016), could be indicative of specific pathophysiological processes rather than a byproduct of methodological variation.

The role of inhibitory circuits in focal seizure initiation and speard has been studied in an acute rodent model (Liou et al., 2018). Liou et al. (2018) observed that ictal neuronal bursts were confined to a 2–3 mm region, but these bursts were accompanied by increased interneuron activity outside the seizure focus, suggesting a protective role of inhibitory circuits in limiting seizure spread. When inhibition was disrupted by applying bicuculline, a GABA-A receptor antagonist, the propagation of seizures became more contiguous, highlighting the critical role of intact inhibitory mechanisms in shaping seizure dynamics. Consistent with these findings, we observed that in a subset of patients who exhibited high inflow of information towards SOZ during the preictal period, non-SOZ regions appeared to exert regulatory control over the SOZ, potentially acting to prevent or delay the transition to the ictal state. In contrast, in another group of patients, the SOZ exhibited greater outflow compared to non-SOZ regions, suggesting that the SOZ had already begun to exert control, potentially driving the transition into seizure onset. It is important to note that these estimates were derived from a 28-s window preceding seizure onset, and the directional flow dynamics between SOZ and non-SOZ regions may vary at different timescales or stages of the preictal period.

From a Network Physiology perspective, our findings suggest that seizure generations are governed by dynamic shifts in information flow within the brain’s functional network. The observed preictal connectivity patterns—characterized by increased inflow of information to SOZ in some patients and high outflow from SOZ to the rest of the network in others—highlight the role of network reorganization in epileptic transitions. These results support the notion that epilepsy is not merely a focal disorder but a network-level phenomenon, reinforcing the importance of applying Network Physiology concepts to better understand seizure dynamics and improve localization strategies for surgical intervention.

## 5 Limitations and future directions

While our study provides valuable insights into preictal connectivity patterns, several limitations should be acknowledged, including variability among patients in epileptic focus localization, etiology, and implantation strategies. Additionally, a significant limitation of this study arises from the differing data structures across the three datasets. While the CHUM dataset provided seizure onset channels for each seizure, the two open-access datasets provided seizure onset channels at a patient level. This disparity may have contributed to the observation that a larger proportion of CHUM patients could be classified into either the inflow or outflow scenarios, as their seizure onset data was more specific. Furthermore, the lack of follow-up time points used for Engel classification across all datasets prevented a direct comparison between them.

Additionally, incorporating high-frequency oscillations (HFOs) and slow-wave activity into the analysis could provide more comprehensive insights into how specific frequency bands interact with connectivity measures. This would allow for a more nuanced classification of seizure onset patterns and potentially lead to the identification of novel biomarkers for predicting seizure onset across different patient populations. Future studies should also consider increasing the sample size and exploring machine learning approaches to classify seizure onset patterns based on both frequency and connectivity measures, to enhance the reliability of these findings and support clinical applications.

One limitation of this study is the absence of a baseline interictal period for comparison. Since seizure-free intervals were not consistently available across all datasets, we were unable to evaluate how the observed preictal connectivity patterns differ from interictal activity. Including interictal data could provide a clearer distinction between pathological and normal connectivity dynamics, offering deeper insights into seizure onset mechanisms. Future studies should aim to incorporate interictal periods to strengthen the interpretation of preictal connectivity changes.

Another limitation of this study is the lack of information regarding the exact distances between implanted electrodes (Supplementary Figures S4, S5). Electrode spacing can influence connectivity estimates, potentially affecting the observed inflow and outflow patterns (Wang et al., 2020). Future studies with precise electrode localization data could further clarify the impact of spatial distribution on network measures.

Finally, it is important to acknowledge the lack of consensus in the literature regarding the duration of the preictal period. In this study, we defined the preictal period as the 28 s preceding seizure onset. This duration was chosen to ensure a consistent preictal period length across patients from multiple centers. While our analysis of this 28-s window identified two distinct patterns of interactions between SOZs and non-SOZs, we did not investigate the dynamics of inflow and outflow beyond this time frame.

## Data Availability

The datasets ds003029 and ds004100 for this study can be found in the https://openneuro.org/datasets/ds003029/versions/1.0.7 and https://openneuro.org/datasets/ds004100/versions/1.1.3, respectively. The CHUM dataset is not publicly available, as the sharing of human data is subject to approval by CHUM’s Research Ethics Board.
