# Supplementary material for: Preictal connectivity dynamics: Exploring inflow and outflow in iEEG networks
Source: Front Netw Physiol. 2025 Apr 28;5:1539682. doi: 10.3389/fnetp.2025.1539682 (PMC12067418; doi:10.3389/fnetp.2025.1539682)
Supplement: Supplementary file 1 [file DataSheet1.pdf]

Supplementary Material

1 Supplementary Figures and Tables

1.1 Supplementary Figures

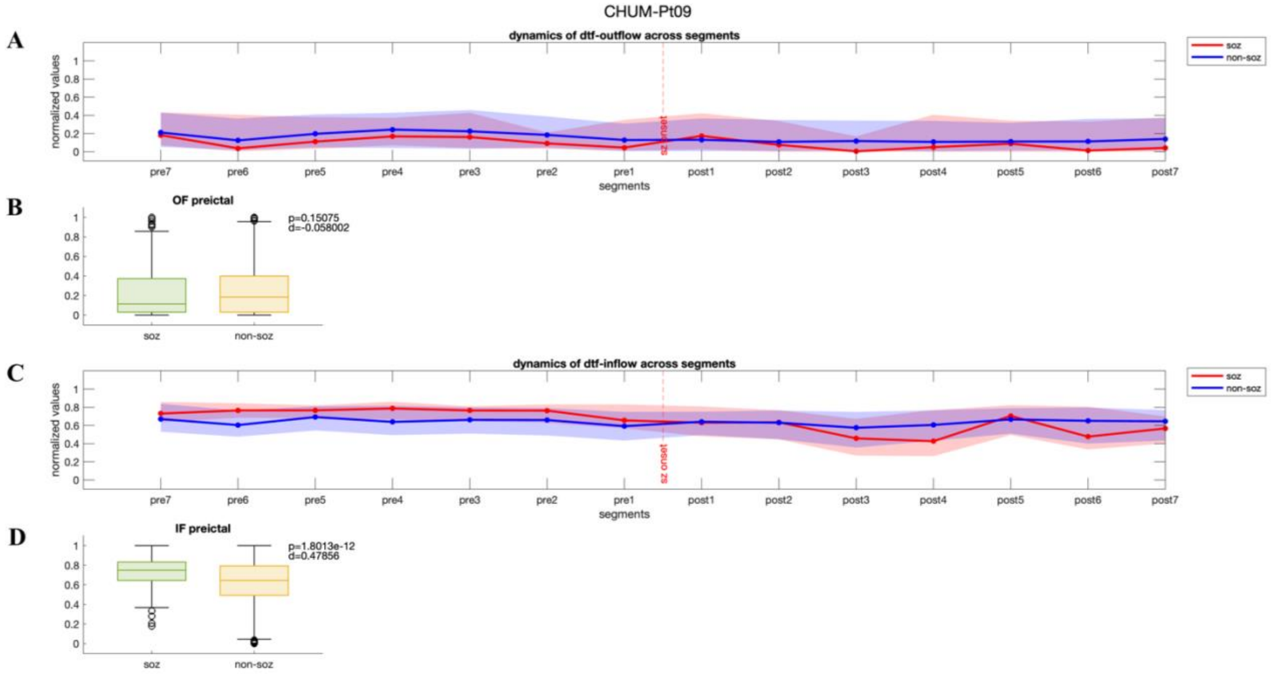

Figure 1. (**A and C**) Dynamics of DTF-based inflow and outflow for SOZ and non-SOZ channels over 28 seconds during the preictal and ictal periods. (**B and D**) Although no significant difference is observed in the median outflow between SOZ and non-SOZ channels (p = 0.15, Wilcoxon rank-sum test; Cohen d effect size=0.051), the median inflow to SOZ channels is significantly higher than that of non-SOZ channels (p = 1.8013e-12; Cohen d effect size=0.49). IF: inflow; OF: outflow

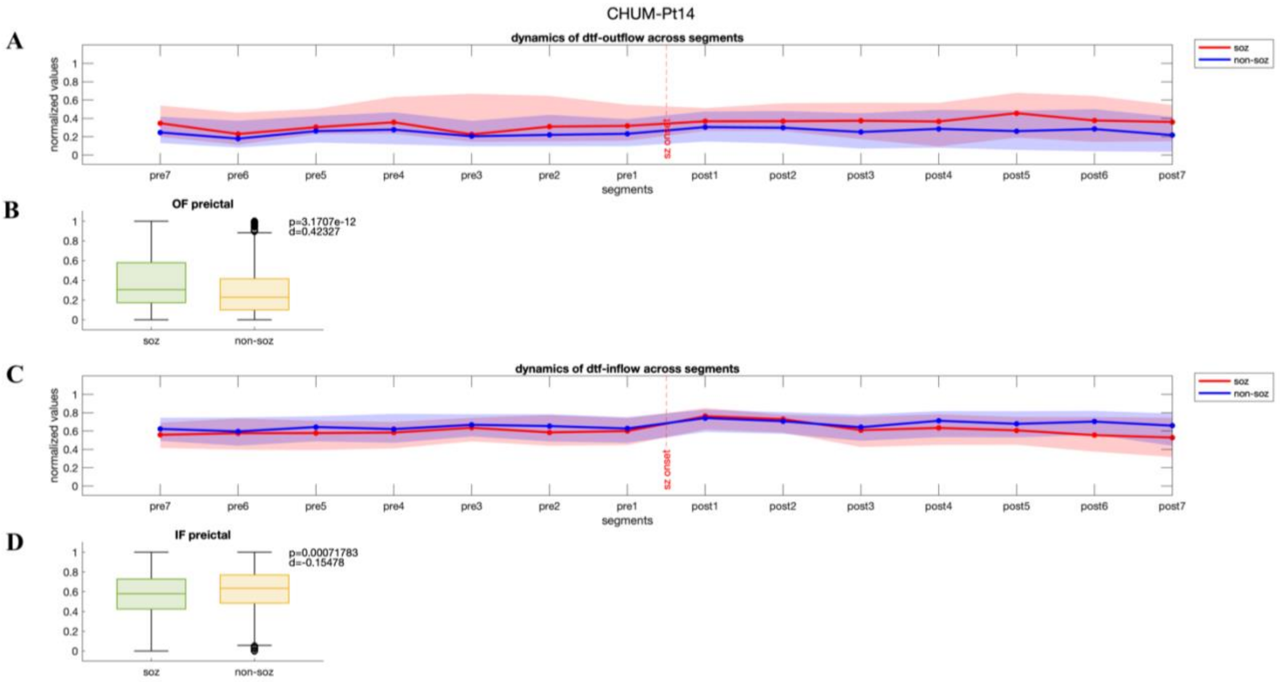

Figure 2. (**A and C**) Dynamics of DTF-based inflow and outflow for SOZ and non-SOZ channels over 28 seconds during the preictal and ictal periods. (**B and D**) Statistical analysis reveals that the median outflow from SOZ channels is significantly higher than from non-SOZ channels (p = 3.17e-12, Wilcoxon rank-sum test; Cohen's d effect size=0.42), while the median inflow to non-SOZ channels is significantly higher than to SOZ channels (p = 0.00072, Wilcoxon rank-sum test; Cohen's d effect size=0.15). IF: inflow; OF: outflow

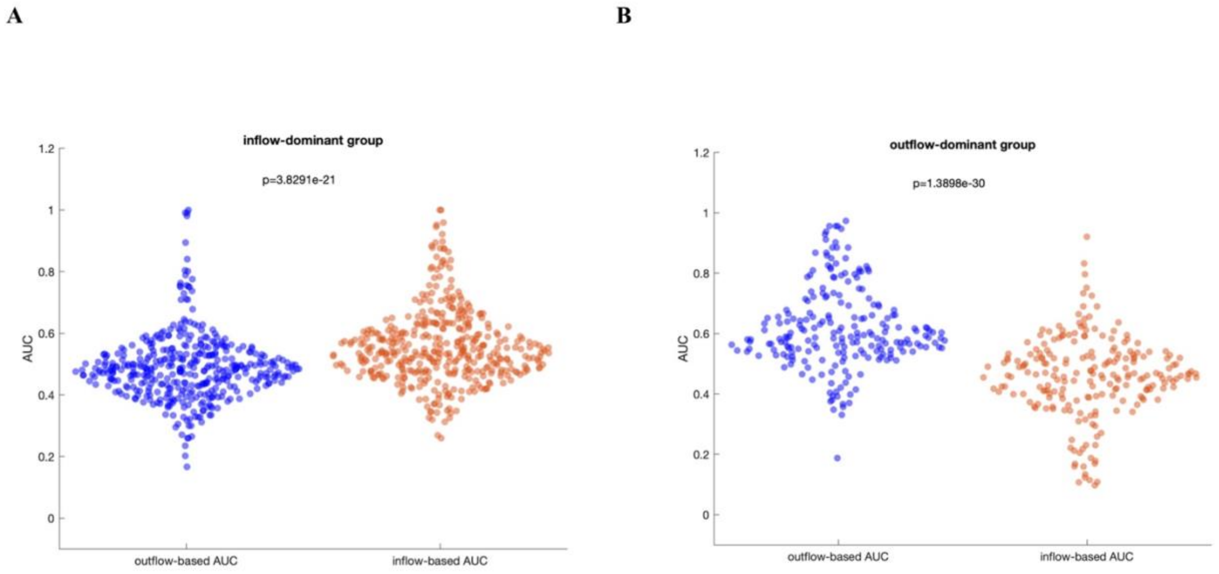

Figure 3. (**A**) Inflow-dominant group: In this group, the median AUC values based on inflow are significantly higher than those based on outflow (p = 3.83e-21). (**B**) Outflow-dominant group: In this group, the median AUC values based on outflow are significantly higher than those based on inflow (p = 1.39e-30, Wilcoxon rank-sum test).

The influence of electrode distance on inflow/outflow values:

While previous studies have shown an inverse correlation between distance and the strength of connectivity measures (Shi et al., 2024, Brain Commun), our analysis revealed patient-specific results (Supplementary Figure 4). When pooling the results across all patients, we found that functional connectivity remained relatively constant as a function of distance (Supplementary Figure 5). This discrepancy may be attributed to several factors that differ between our study and previous work. The choice of reference montage in iEEG recordings can have a significant impact on the measured connectivity, especially at short and long distances. As demonstrated by Shi et al. (2024), common average referencing, which we used in our study, tends to reduce spurious correlations at short distances more effectively than bipolar referencing. Bipolar montage, commonly employed in prior studies (Lagrade et al., 2018, Brain), has been shown to be less effective in minimizing such correlations.

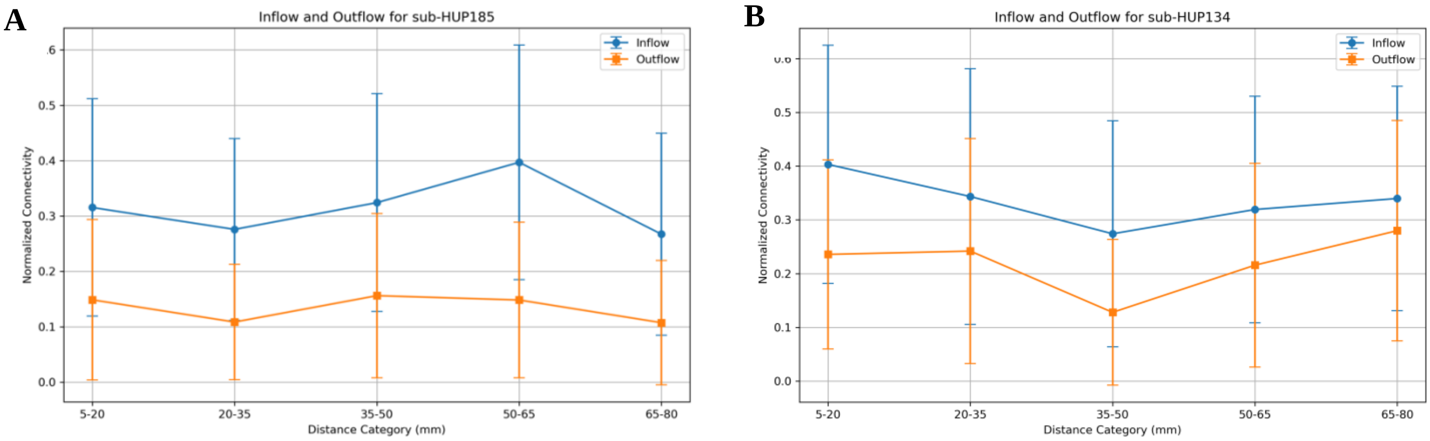

Figure 4. Inflow and outflow values as a function of 5 distance groups. A and B are two patients with different influence of distance.

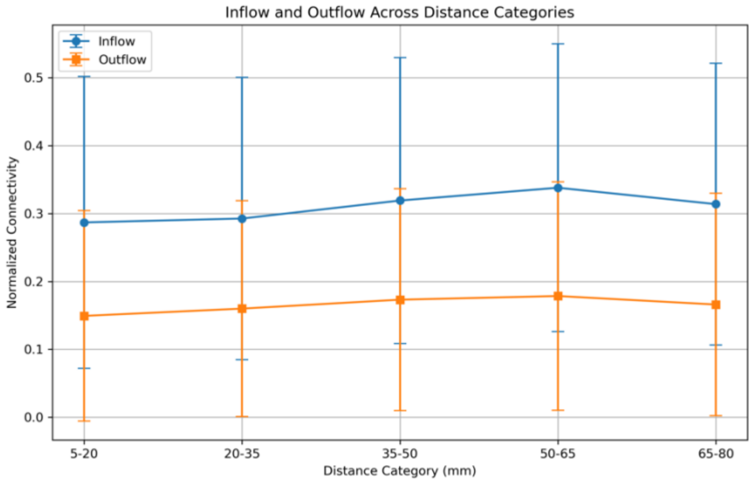

Figure 5. Pooled inflow/outflow values as a functional of 5 distance categories.

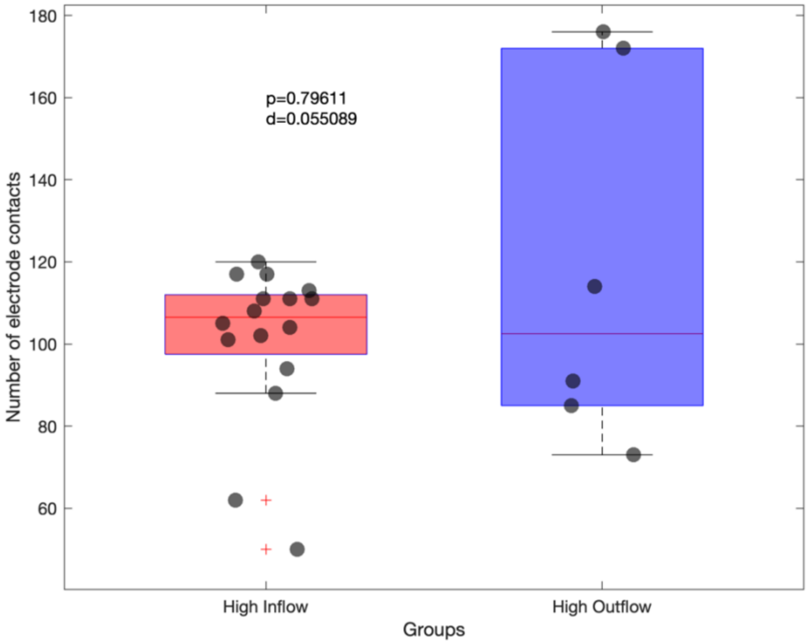

Figure 6. Influence of the number of implanted electrodes on our findings. Statistical analysis showed that there is no significant difference between high-inflow and high-outflow groups (p=0.80, Wilcoxon rank-sum test; effect size is computed by Cohen's d = 0.055).

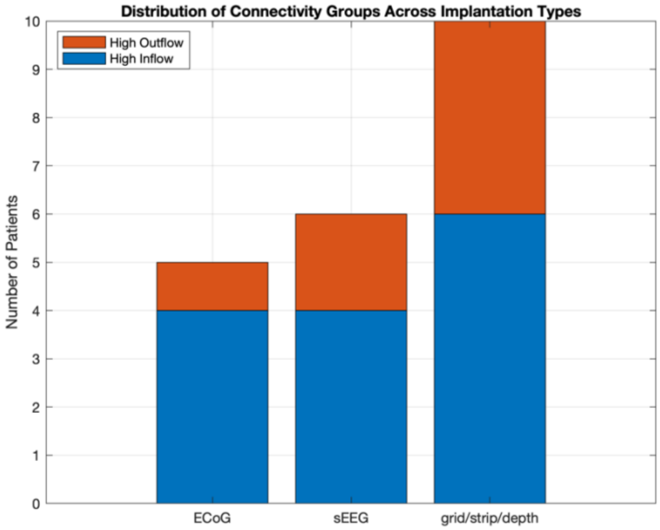

Figure 7. Number of patients classified in each observed groups (i.e., high inflow and high outflow) with respect to the implantation approaches (i.e., ECoG, SEEG, and a combination of grid/strip/depth electrodes).

A **B**

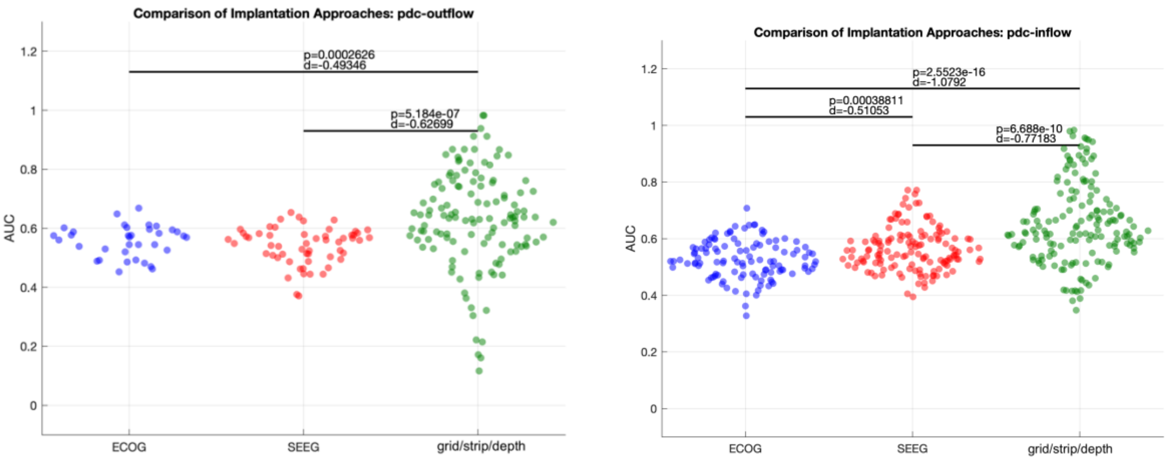

Figure 8. AUC values across the three implantation approaches (ECoG, sEEG, and a combination of grid/strip/depth electrodes) extracted from PDC-based measures. (**A**) High-outflow group, (**B**) High-inflow group. P values are computed with Wilcoxon rank-sum test and the effect size is computed by Cohen d metric. Statistical analysis showed that patients with a combined grid/strip/depth implantation exhibited significantly higher AUC values than those with either ECoG or sEEG alone in both groups.

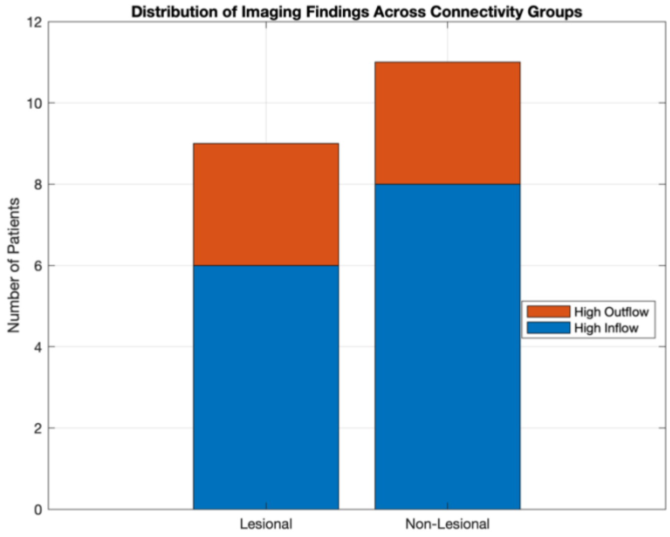

Figure 9. Number of classified patients in each category (i.e., high inflow and high outflow) with respect to MRI findings (i.e., lesional vs. non-lesional).

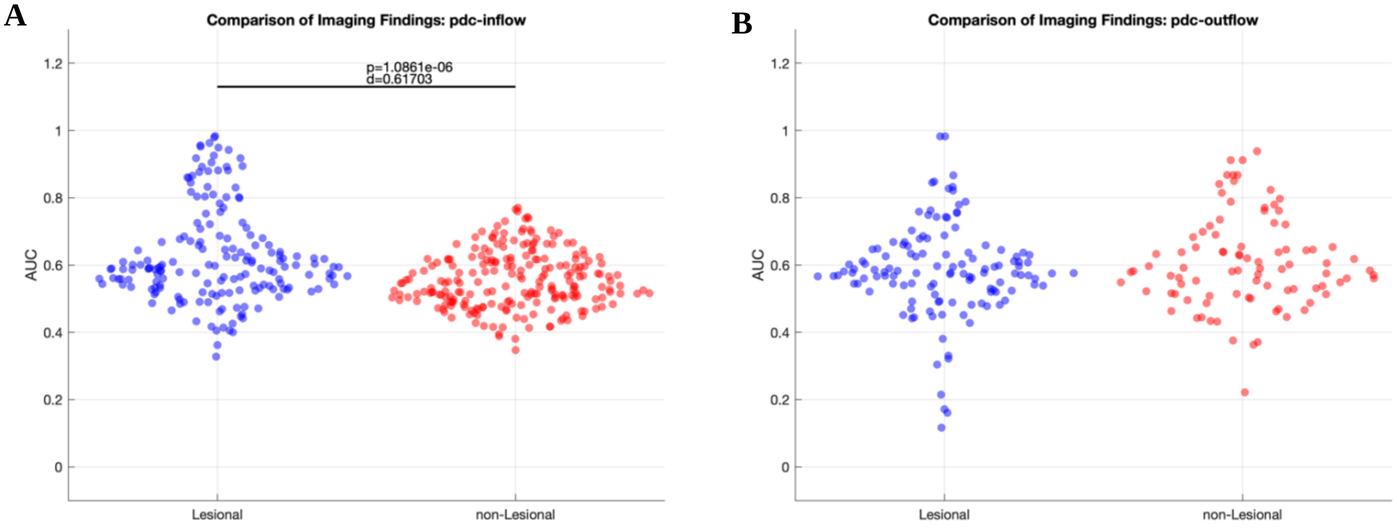

Figure 10. Statistical comparison between lesional and non—lesional groups of patients. (**A**) High inflow group, (**B**) High outflow group. P values are computed with Wilcoxon rank-sum test and the effect size is computed by Cohen d metric. Statistical analysis showed that lesional patients showed significantly higher AUC values in high inflow group (p=1.086e-06, d=0.62). However, no significant difference was observed between lesional and non-lesional patients in high outflow group.
